# First Experience of Unlocking The "MIstery" in Benign Prostatic Obstruction in Nepal: A Case Report

**DOI:** 10.31729/jnma.v63i2091.9239

**Published:** 2025-11-30

**Authors:** Subodh Ghimire, Mahesh Bahadur Adhikari, Bipin Maharjan, Ravi Kiran Gautam, Ajit Khadga

**Affiliations:** 1 Department of Urology and Renal Transplant, Nepal Mediciti

**Keywords:** *Benign prostatic obstruction*, *LUTS*, *Minimally Invasive Surgical Therapy (MIST)*, *UroLift*

## Abstract

Benign prostatic obstruction is a prevalent cause of lower urinary tract symptoms in aging men, traditionally managed with medical therapy or invasive surgery. Minimally invasive surgical therapies like the UroLift® system have gained global recognition for their efficacy and safety, yet their adoption in low-resource settings has been limited. We present the first documented case of UroLift performed in Nepal on a 73 years old gentleman with persistent lower urinary tract symptoms unresponsive to dual therapy. The procedure was completed successfully without complications, resulting in significant symptom relief and rapid postoperative recovery. This milestone highlights the feasibility of introducing advanced minimally invasive surgical therapy option like UroLift in Nepal, offering a promising alternative to medicines or surgery for benign prostatic obstruction.

## INTRODUCTION

Lower Urinary Tract Symptoms (LUTS) associated with Benign Prostatic Hyperplasia (BPH) is increasingly prevalent condition in ageing men and it at times can significantly affect their daily life.^1^ The LUTS due to BEP is initially manged with medical therapy followed by surgery either monopolar or bipolar Transurethral Resection of Prostate (TURP) in certain cases only.^2^ In recent years, different techniques have been developed aiming to reduce short- and long-term surgical side effects and with the intent to be minimally invasive.^2^ We share the nation first experience of minimally invasive treatment for benign enlargement of prostate in this report.

## CASE REPORT

A seventy-three-year-old gentleman with a comorbidity of Chronic obstructive pulmonary disease with domiciliary oxygen therapy, with a year-long dual therapy for LUTS due to BEP, presented to our clinic with complaints of poor flow of urine, a sensation of incomplete voiding, nocturia, straining to pass urine, hesitancy, and intermittency of urinary stream. On objective evaluation, his International Prostate Symptom Score was 19, and he was very unhappy with his quality of life, with a score of 5. He had a good performance status with an Eastern Cooperative Oncology Group (ECOG) performance score of 2. His vital functions were stable except for the need for oxygen.

He had a small, benign prostate on digital rectal examination. Further evaluation with ultrasonography of the abdomen revealed a 40-gram prostate without intravesical protrusion. His uroflowmetry showed that for a voided volume of 180 ml, his maximum flow (Q max) was 11.8 ml/sec, average flow (Q avg) was 2.7 ml/sec, and he had a high post-void residual urine of 165 ml. Cystoscopy revealed bilobar enlargement of the prostate, causing luminal obstruction without intravesical protrusion.

In this gentleman, the medicines weren’t doing well for his urinary symptoms, and TURP was not an option, considering his domiciliary oxygen therapy and inability to lie supine completely. We had to look for a midway in between the surgery and drug therapy, and considering the size of the prostate without the intravesical protrusion, we gave him the option of prostatic urethral lift or UroLift. He and his family members were informed about the pros and cons of the procedure, and after their agreement, the procedure was done.

**Figure 1:**
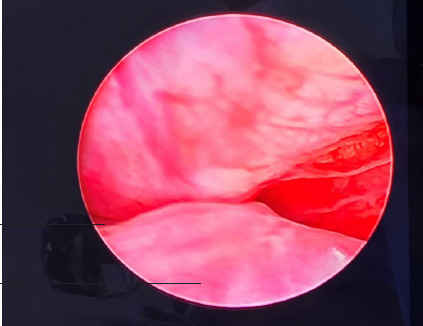
Cystoscopic view showing occluding prostate.

The procedure was done under local anesthesia with a full tube of 2% lignocaine jelly instilled into the urethra, and after waiting for 5 minutes cystoscopy was done and the obstruction done by the enlarged prostate was observed (Figure 1) and then the UroLift system was inserted under cystoscope guidance and four implants were deployed to retract the obstructing lobes of prostate away from the urethra.

**Figure 2:**
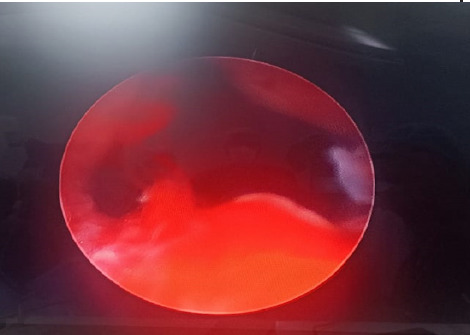
Opening of the urethral lumen after four implants.

At last, the urethral opening was wide open with the retracted lobes of the prostate (Figure 2). He was sent home on the same day with a foley catheter and a short course of antibiotics and an alpha blocker, and was reviewed after 5 days, and his catheter was successfully removed. He came for follow-up after a month, and his IPSS score decreased to 9 from 19, and he was mostly satisfied with the quality of life, with a score of 2, and Uroflowmetry done at this time showed that for a voided volume of 320ml, Qmax was 26.2ml/sec, Q avg 10.7ml/sec, and PVRU decreased to 99 ml. He is currently on all the medicines for urinary symptoms and is scheduled for follow-up after 3 months for further evaluation.

## DISCUSSION

Benign Prostatic Hyperplasia is a condition in which the prostate enlarges and causes a group of different storage and voiding symptoms, which are collectively known as LUTS.^3,4^ As a first line of treatment, the management begins with medical therapies such as alpha-adrenergic antagonists to relax the bladder neck and 5-alpha reductase inhibitors to decrease the size of the prostate.^5^ Among the patients treated with medicines, a significant group fails with the medical therapy, and various option of surgical interventions are available for managing symptoms and improving quality of life.^6^

Transurethral Resection of Prostate with the use of monopolar or bipolar is the current gold standard for patients with bothersome moderate-to-severe LUTS secondary to benign prostatic obstruction (BPO).^7^ In recent years, different techniques have been developed aiming to reduce short- and long-term surgical side effects, and with the intent to be minimally invasive, and are collectively known as Minimally Invasive Surgical Therapy (MIST).^2^ MISTs are considered an intermediate step between BPH medication and conventional surgery, and they are safe and characterized by low intraoperative and postoperative complications.^2^ The essential characteristics of these MISTs are feasibility in outpatient and one-night stay settings, minimal bleeding risk, early postoperative recovery of daily activities, and a reduced impact on urinary continence and ejaculatory function.^8^

Among various options of MISTs, the UroLift procedure also known as Prostatic Urethral Lift is undertaken transurethrally by using a pre-loaded delivery device which is passed through a rigid sheath under cystoscopic visualisation. The delivery device is used to compress one lateral lobe of the prostate towards the prostatic capsule after which a needle is used to deploy the implant, with one end of the implant anchored in the urethra and the other on the outer surface of the prostatic capsule, retracting the prostatic lobe away from the urethral lumen and usually multiple implants are used during each procedure.^9^

The first multicentre randomised blinded trial of prostatic urethral lift for the treatment of lower urinary tract symptoms secondary to benign prostatic hyperplasia was done by Roehrborn CG et al. including more than 200 patients. The patients were followed for a year long duration. The authors concluded that the prostatic urethral lift, can be reliably performed with the patient under local aesthesia, provides rapid and sustained improvement in symptoms and flow, while preserving sexual function.^10^

A systematic review and meta-analysis including more than a 30 studies about the rate of reintervention of newer surgical interventions have concluded that that the re-intervention rate with these new surgical interventions is comparable if not better than TURP in the short term and it remains an important factor for consideration when choosing one form of surgical intervention over another novel therapy. The clinicians should be aware of the existing limitations of each new surgical device and longer-term data on these novel therapeutic options is needed so that patients can be adequately informed regarding their choice in a shared decision-making process with their surgeon.^11^

With an increasing aging population, the burden of LUTS due to BEP will be rising. A significant proportion of elderly individuals will need some form of intervention for bothersome urinary symptoms. UroLift represents a feasible option as a bridge between medical therapy and surgical intervention with an acceptable outcome and minimal complications. Although it is based on a single case and we cannot generalize the outcome to all patients, as the case selection is the most important factor for a good outcome in UroLift, this paper highlights the milestone of feasibility in introducing advanced MIST options, such as UroLift, in Nepal, offering a promising alternative to medication or surgery for benign prostatic obstruction.
